# Vereinbarkeit von Pflege bei Demenz, Familie und Beruf

**DOI:** 10.1007/s00391-020-01764-9

**Published:** 2020-07-27

**Authors:** Lydia Neubert, Sophie Gottschalk, Hans-Helmut König, Christian Brettschneider

**Affiliations:** Institut für Gesundheitsökonomie und Versorgungsforschung, Hamburg Center for Health Economics, Universitätsklinikum Hamburg-Eppendorf, Martinistraße 52, 20246 Hamburg, Deutschland

**Keywords:** Angehörigenpflege, Doppelbelastung, Beziehungsqualität, Aufgabenverteilung, Typologie, Informal care, Double burden, Relationship quality, Task distribution, Typology

## Abstract

**Hintergrund:**

Pflegende Angehörige (PA) von Menschen mit Demenz (MMD) sind eine vulnerable Personengruppe, die nicht nur mit den Belastungen aufgrund der Pflege, sondern auch mit Anforderungen aus ihrem Familien- und Berufsleben konfrontiert sein können. Der nationale Forschungsstand zum Erleben des Spannungsfelds zwischen Pflege, Familie und Beruf ist unzureichend.

**Ziel der Arbeit:**

Die vorliegende Studie ist eine rekonstruktive Analyse der Vereinbarkeit der Pflege eines MMD mit den Lebensbereichen Familie und Beruf, in der Haupt- und Nebenpflegende des MMD berücksichtigt wurden.

**Methoden:**

Es wurden 14 narrative Interviews mit PA von MMD geführt. Die Auswertung erfolgte anhand der Dokumentarischen Methode nach Nohl und mündete in Typenbildungen.

**Ergebnisse:**

In pflegenden Familien eines MMD bedingen sich Beziehungsqualität und Aufgabenverteilung gegenseitig. Dies hat Auswirkungen auf das Erleben der Pflege. Stabile Beziehungen und fair verteilte Aufgaben können die Pflege erleichtern, wohingegen konfliktbelastete Beziehungen und ungleich verteilte oder ungeteilte Aufgaben die Pflegebelastung der Familie erhöhen. Erwerbstätige PA nehmen die Lebensbereiche Pflege und Beruf als getrennt voneinander, miteinander in Konflikt geratend oder sich gegenseitig unterstützend wahr.

**Diskussion:**

Die Belastungen aufgrund der Pflege eines MMD führen zu Beeinträchtigungen im Familien- und Berufsleben, was negative Folgen für die Gesundheit der PA haben kann. Doch ebenso können die Familie sowie der Beruf die Belastung reduzieren, wenn PA beispielsweise durch therapeutische Angebote darin unterstützt werden, positive Pflegeerfahrungen wie gestärkte Familienbeziehungen und -funktionalität zu erleben, und wenn erwerbstätige PA den Beruf als einen stärkenden Lebensbereich erfahren.

**Zusatzmaterial online:**

Zusätzliche Informationen sind in der Online-Version dieses Artikels (10.1007/s00391-020-01764-9) enthalten.

## Abstract (en)

**Background:**

Informal caregivers of people with dementia (PWD) are a vulnerable group, which is not only confronted with potential burdens due to caring but also with the demands of the family and working life. The state of national studies on experiencing the tensions between care, family and work is insufficient.

**Objective:**

This qualitative study is a reconstructive analysis of the compatibility between caregiving with the areas of family and working life, in which the primary and secondary caregivers of the PWD were taken into account.

**Methods:**

We conducted 14 narrative interviews with family caregivers of PWD. The analysis was based on Nohl’s documentary method and led to the formation of different types.

**Results:**

In caring families of PWD, subjective relationship quality and task distribution are mutually dependent and this affects the family’s care experiences. Stable relationships and fairly distributed tasks can facilitate caregiving, whereas conflict-ridden relationships and unfairly distributed or undivided tasks increase the family’s care burden. Employed caregivers perceive the areas of life, caregiving and work as separate, conflicting or mutually supportive.

**Conclusion:**

The burden of informal caregiving for PWD leads to impairments in family and working life, which can have negative consequences for the health of the family caregivers; however, family and work can also reduce the burden if caregivers are supported by, for example therapeutic services, to experience positive care experiences, such as strengthened family relationships and functionality and if employed caregivers experience work as a strengthening area of life.

Pflegende Angehörige (PA) von Menschen mit Demenz (MMD) sind nicht nur informelle Pflegepersonen der hilfebedürftigen Verwandten, sondern begegnen auch Erwartungen, die ihre Familien beispielsweise als Ehepartner, Elternteil oder Geschwister an sie stellen. Wenn sie erwerbstätig sind, sind sie zusätzlich mit den Anforderungen aus ihrem Berufsalltag konfrontiert. Dieser Beitrag beschreibt die Sicht von PA auf das Spannungsfeld zwischen Pflege, Familie und Beruf.

## Hintergrund und Fragestellung

Die Zahl der MMD nimmt international wie national kontinuierlich zu [8]. Viele von ihnen können dank ihrer PA lange zu Hause leben. In Deutschland ist der Anteil von Pflegebedürftigen, die allein durch Angehörige versorgt werden, in den letzten Jahren deutlich angestiegen [26]. Insbesondere PA von MMD sind – aufgrund der besonderen Anforderungen, die mit dem Erkrankungsbild (z. B. herausfordernde Verhaltensweisen) einhergehen – stark belastet [5, 14], und neben der informellen Pflege können auch andere Lebensbereiche wie die Familie und der Beruf hohe Anforderungen an sie stellen.

Das bis heute viel benutzte Modell zur Erklärung von pflegebedingtem Stress von Pearlin et al. [22] beschreibt die Familie als wichtigen Teil des informellen Netzwerks (NW) von PA. Dabei kann sie sowohl Ressource (z. B. in Form von praktischer Unterstützung) als auch Stressor (z. B. bei Unstimmigkeiten über die Pflege) sein [18, 28]. Im Vergleich zu Pflegebedürftigen ohne Demenz stehen hinter MMD größere informelle NW und dadurch mehrere Pflegende, die sich die Aufgaben teilen [25]. Dennoch können sich (Haupt‑)Pflegende durch Angehörige, die sich nicht oder zu wenig an der Pflege beteiligen, zusätzlich belastet fühlen [1].

Immer mehr Angehörige, die Unterstützung für ältere Verwandte leisten, vereinbaren dies mit ihrer Erwerbstätigkeit [15]. Der Zusammenhang zwischen informeller Pflege eines MMD und Erwerbstätigkeit der PA ist überwiegend negativ; häufig kommt es zu Arbeitszeitreduktion, Fehlzeiten, Arbeitsunterbrechungen, verminderter Arbeitsleistung und Karriereeinbußen [2]. Einerseits können die Doppelbelastung und Vereinbarkeitsprobleme die Gesundheit von PA beeinträchtigen [30], andererseits kann der Beruf Erholung von der Pflege ermöglichen und das Wohlbefinden der PA fördern [9, 13].

Zusammenfassend betrachtet befinden sich viele PA von MMD in einem Spannungsfeld, das aus den gleichzeitigen Anforderungen aufgrund der Pflege, der Familie und des Berufs resultiert. Dies kann in der Folge auch zu hoher Belastung und gesundheitlichen Beeinträchtigungen führen. Diese Mixed-methods-Pilotstudie soll den nationalen Forschungsstand zum Thema Vereinbarkeit von informeller Pflege, Familie und Beruf ergänzen, indem sie unter Einnahme einer Netzwerkperspektive die Handlungspraktiken von betroffenen PA im Spannungsfeld von Pflege, Familie und Beruf ergründet. Anhand der forschungsleitenden Fragen „Wie nehmen pflegende Angehörige a) das Familienleben und b) ihre Erwerbstätigkeit unter der Pflege wahr?“ wurden Haupt- und Nebenpflegende zu ihrem Erleben befragt und ihre Handlungspraktiken rekonstruiert.

## Studiendesign und Untersuchungsmethoden

Der vorliegende Artikel gibt die Ergebnisse des qualitativen Forschungsstrangs einer Mixed-methods-Pilotstudie [3] wieder (*QUAL+quan design*; weitere Informationen im Zusatzmaterial online: Supplement 1). Die eingeschlossenen NW bestanden aus PA und, wenn vorhanden, aus nichtverwandten Pflegenden (z. B. Ehrenamtliche) von zu Hause lebenden MMD aus Nord- und Süddeutschland. Die Interviews fanden im Zeitraum vom September 2017 bis September 2018 statt. Die Interviews mit den Mitgliedern eines in Süddeutschland lebenden NW wurden per Skype geführt. Alle anderen Interviews führte Untersucherin 1 im eigenen Zuhause der TeilnehmerInnen (Norddeutschland). Die Tonaufnahmen wurden pseudonymisiert transkribiert. Einschlusskriterien, Sampling-Strategie und Rekrutierungswege erfolgten bis auf 2 Abweichungen wie im Studienprotokoll [3] vorgesehen. In 2 NW konnten nur 2 anstelle der vorgesehenen 3 TeilnehmerInnen gewonnen werden, weswegen letztendlich 7 anstatt 5 NW eingeschlossen wurden. Ein NW trat über einen Kontakt auf einer Konferenz in die Studie ein. Alle anderen NW traten, wie vorgesehen, über die Vermittlung durch Gatekeeper (z. B. BeratungsstellenmitarbeiterInnen) in die Studie ein.

Zur Datenerhebung wurden narrative Interviews [24] genutzt. Der Erzählstimulus für die narrativen Interviews lautete „*Wie Sie ja wissen, interessiere ich mich dafür, wie das mit der Pflege [Ihrer/Ihres …] in Ihrer Familie läuft. Aber um das Ganze einordnen zu können und um zu verstehen, wie das angefangen hat, bitte ich Sie nun, mir die Geschichte Ihrer Familie bis zum heutigen Zeitpunkt zu erzählen*“. Das Thema *Vereinbarkeit von Pflege und Familie* wurde von beinahe allen TeilnehmerInnen im Verlauf der Interviews ohne weitere Erzählaufforderung angesprochen. Die *Vereinbarkeit von Pflege und Beruf* musste überwiegend nachgefragt werden (hierzu enthielt der Interviewleitfaden erzählgenerierende Formulierungen, die je nach bereits Berichtetem angepasst wurden).

Die Interpretation der Interviews erfolgte anhand eines rekonstruktiven Vorgehens, der Dokumentarischen Methode nach Nohl [20, 21]. Das Vorgehen verläuft in mehreren Schritten. Untersucherin 1 und Untersucherin 2 führten die formulierende und reflektierende Interpretation für ausgewählte Interviewpassagen einzelner Fälle getrennt voneinander durch. Ergebnis hiervon sind Beschreibungen, in denen die dem jeweiligen Fall eigenen Handlungspraktiken (Dimensionen und Orientierungsrahmen) hinsichtlich des Erkenntnisinteresses rekonstruiert wurden. Die Schritte Fallvergleich und Typenbildung durchliefen Untersucherin 1 und Untersucherin 2 gemeinsam und diskutierten die Ergebnisse mit Untersucher 3. Letztendlich liegen fallübergreifende Orientierungsrahmen vor, in denen Handlungs- und Haltungstypen rekonstruiert wurden: In der Interpretation zur *Vereinbarkeit von Pflege und Familie* gelang eine relationale Typenbildung (bestehend aus 5 Typen, die aus den regelmäßigen Verbindungen zwischen den Orientierungsrahmen zweier Dimensionen heraus entstanden sind). Die Interpretation zur *Vereinbarkeit von Pflege und Beruf* endete in einer sinngenetischen Typenbildung (bestehend aus 3 Typen). Zusätzlich wurde das Textmaterial regelmäßig in Interpretationswerkstätten konsensuell validiert.

Im Anschluss an die Interviews füllten die TeilnehmerInnen NW-Karten aus, die die Struktur des jeweiligen NW (Akteure und Verbindungen unter ihnen) widerspiegeln. Die NW-Karten einzelner NW-Mitglieder wurden für das jeweilige (Familien‑)NW zu einer gemeinsamen NW-Karte kombiniert. Diese NW-Karten wurden im Team und im Rahmen einer Interpretationswerkstatt anhand der „qualitative structural analysis“ [12] interpretiert. Zusammengefasst geht es hierbei darum, Fragen an die NW-Karten zu stellen (ohne im besten Fall die Interviews zu kennen), auf die das Interviewmaterial Antworten liefern kann. Zudem offenbarten die NW-Karten Zusammenhänge bzw. Verbindungen, die aus dem Textmaterial nicht rekonstruiert werden konnten (Zusatzmaterial online: Supplement 1). Bedingt durch die eingenommene NW-Perspektive sind die Fälle eines NW unter Berücksichtigung ihres jeweiligen Kontextes, aber damit auch abhängig voneinander rekrutiert worden. Diesem Umstand begegnet die komparative Sequenzanalyse [20], die die Loslösung von der NW-Zugehörigkeit der Einzelfälle ermöglicht. In der formulierenden und reflektierenden Interpretation wurden Orientierungen rekonstruiert, zu denen durch Fallvergleiche Anschlussäußerungen aus anderen Fällen gesucht wurden. Die so rekonstruierten Orientierungsrahmen und Dimensionen umfassen nicht nur die Orientierungen eines Falles, sondern die Orientierungen verschiedener Fälle. Dabei müssen die tatsächlichen Fälle eines NW nicht einen Typus bilden (kam aber bei sehr ähnlichen Orientierungen vor), sondern finden sich in verschiedenen Typen wieder. Die komparative Sequenzanalyse ermöglicht und erleichtert nicht nur den interpretatorischen Zugriff, sondern dient auch zur Validierung der Interpretation, indem die dadurch erreichte methodische Kontrolle und Reflexion der Standortgebundenheit der Forschenden (z. B. in Form des Wissens über die NW-Zugehörigkeit der Einzelfälle) entgegenwirkt [20, 21].

## Ergebnisse

Insgesamt nahmen 7 NW mit 19 informell Pflegenden von zu Hause lebenden MMD an der Studie teil. Darunter waren 14 PA, deren Interviews Grundlage für die Interpretation zur *Vereinbarkeit von Pflege und Familie* sind. Die Interpretation zur *Vereinbarkeit von Pflege und Beruf* beruht auf 11 Interviews mit den zum Untersuchungszeitpunkt erwerbstätigen PA. Das Durchschnittsalter der Stichprobe beträgt 48,8 Jahre, und die meisten TeilnehmerInnen sind die erwachsenen Kinder der MMD. Alle familiären NW erhalten Unterstützung durch ambulante Pflegedienste, 5 NW von ehrenamtlichen oder selbstständigen Betreuungskräften und 2 NW beschäftigen eine 24-h-Betreuungskraft. Die TeilnehmerInnen auf Individual- und NW-Ebene beschreiben Tab. 1 und 2.

**Table 1:** 

	Durchschnitt	Spannweite	*n* (%)
*Pflegende Angehörige (n* *=* *14)*			
*Alter (Jahre)*	48,79	19–65	–
*Weiblich*	–	–	10 (71,4)
*Verwandtschaftsverhältnis zum MMD*			
Ehepartner(in)	–	–	1 (7,1)
Erwachsenes Kind	–	–	9 (64,3)
Enkelkind	–	–	3 (21,4)
Schwiegertochter	–	–	1 (7,1)
*Bildungsabschluss (Mehrfachnennung möglich)*			
Hauptschule	–	–	1 (7,1)
Realschule	–	–	4 (28,6)
Gymnasium	–	–	9 (64,3)
Ausbildung	–	–	10 (71,4)
Studium	–	–	3 (21,4)
Sonstiges	–	–	1 (7,1)
*Erwerbstätigkeit*			
Berufstätig	–	–	12 (85,7)^a^
Sonstiges	–	–	1 (7,1)
Fehlende Angabe	–	–	1 (7,1)
*Gemeinsamer Haushalt mit dem MMD*			
Ja	–	–	6 (42,9)
Nein	–	–	8 (57,1)
*Wohngegend*			
Städtisch	–	–	10 (71,4)
Ländlich	–	–	4 (28,6)

^a^Das Sample zur Typologie zur Vereinbarkeit der Pflege mit dem Beruf enthält 11 erwerbstätige, pflegende Angehörige. Eine Teilnehmerin gab „erwerbstätig“ an, tatsächlich aber war sie zum Interviewzeitpunkt Auszubildende, weswegen sie aus diesem Teil der Analyse ausgeschlossen wurde

**Table 2:** 

NW	Geschlecht und Pflegestatus der Angehörigen (*n* = 14)	Verwandtschaftsverhältnis zum MMD	Erwerbstätigkeit und Beruf^a^ (*n* = 11)
A	w, HP	Tochter	j, Chemielaborantin
	w, NP	Tochter	j, Vermessungstechnikerin
	m, NP	Sohn	j, Pflegeberater
B	w, HP	Tochter	n, nicht zutreffend
	w, NP	Tochter	j, selbstständige Apothekerin
C	w, HP	Tochter	j, Speditionskauffrau
	w, NP	Enkeltochter	n, nicht zutreffend
D	m, HP	Ehemann	j, keine Angabe
	w, NP	Enkeltochter	j, Assistentin im Marketing
E	w, HP	Tochter	j, Assistentin der Geschäftsführung
F	w, HP	Schwiegertochter	j, Erzieherin
	m, NP	Sohn	j, Bankangestellter
	w, NP	Enkeltochter	n, nicht zutreffend
G	m, HP	Sohn	j, Tischler

*m* männlich, *w* weiblich, *NP* Nebenpflegende, *HP* Hauptpflegende (HP haben sich selbst als solche bezeichnet; NP sind Verwandte, die sich verhältnismäßig weniger in die häusliche Pflege einbringen), *j* ja, *n* nein

^a^Der Umfang der Erwerbstätigkeit wurde nicht systematisch erfasst. Aus den Interviews ist aber bekannt, dass die erwerbstätigen HP und NP sowohl in Voll- oder Teilzeit tätig waren

### Vereinbarkeit von Pflege und Familie

Auf die Frage nach der *Vereinbarkeit von Pflege und Familie* konnten 2 Dimensionen mit jeweils 3 Orientierungensrahmen rekonstruiert werden, die sich – beschrieben in 5 Typen – gegenseitig beeinflussen (Tab. 3). Die erste Dimension *intrafamiliäre Beziehungsqualität* beschreibt, wie die PA ihre Beziehungen untereinander wahrnehmen. Die zweite Dimension *intrafamiliäre Aufgabenverteilung *beschreibt, wie sie die Verteilung der pflegerischen Aufgaben auf die verfügbaren Familienmitglieder wahrnehmen. Die Beziehungsqualität entscheidet darüber, wie die Aufgabenverteilung wahrgenommen wird, und umgekehrt, je nachdem wie die Aufgaben verteilt sind, werden die Beziehungen unterschiedlich bewertet. Beides hat Auswirkungen auf die Pflegeerfahrung der Familie. Die Ergebnisse werden an anderer Stelle veröffentlicht [4], deswegen sollen hier lediglich anhand zweier kontrastierender Typen die gefundenen Zusammenhänge verdeutlicht werden (weitere Informationen im Zusatzmaterial online: Supplement 2). In Typ 1 trägt jeder PA zur Pflege des MMD bei. Sie verteilen die Aufgaben – beinahe gleichmäßig und ohne komplexes Aushandeln – nach individuellen Fähigkeiten, Charakterzügen und Rollenbildern. Sie treffen gemeinsam Entscheidungen, können sich aufeinander verlassen und teilen gemeinsame Werte. Die PA stehen sich nahe; die Pflege hat die Beziehungen untereinander sogar noch intensiviert. Das harmonische Verhältnis und die geteilte Pflegeverantwortung zwischen den PA sorgen dafür, dass die häusliche Pflege nahezu als leicht zu bewältigen wahrgenommen wird. In Typ 4 dagegen erwarten die PA von ihren Verwandten, dass sie sich als Nebenpflegende einbringen. Da dies nicht geschieht, sind sie enttäuscht, fühlen sich alleingelassen, ausgenutzt und missverstanden. Die PA gehen jedoch unterschiedlich damit um. Einige kämpfen um die Hilfe aus der Familie, andere scheuen Konflikte und suchen stattdessen verstärkt nach externer Hilfe. Wiederum gemeinsam ist ihnen, dass sie die Beziehungen zu ihren Verwandten abbrechen und die häusliche Pflege als stark belastend erleben. Die Dimensionen, Orientierungsrahmen und relationalen Typen im Überblick zeigt Tab. 3. In jedem Typ befinden sich Hauptpflegende, die sich selbst als solche bezeichnet haben, und Verwandte, die sich verhältnismäßig weniger in die Pflege einbringen.

**Table 3:** 

		Intrafamiliäre Aufgabenverteilung		
		Ungeteilt	Ungleich	Geteilt
**Intrafamiliäre Beziehungsqualität**	Harmonisch	–	–	T1: Gemeinschaftsprojekt (*Wir pflegen gemeinsam*)
	Einverstanden	T2: Kooperation mit externer Hilfe (*Wir haben ein Back-up*)	T3: Kooperation in der Familie (*Wir funktionieren*)	–
	Konfliktär	T4: Enttäuschung (*Wir sind enttäuscht*)	T5: Zwangssituation (*Wir haben keine Alternative*)	–

### Vereinbarkeit von Pflege und Beruf

Auf die Frage nach der *Vereinbarkeit von Pflege und Beruf* konnten 3 typisierte Haltungsorientierungen von erwerbstätigen PA rekonstruiert werden, die ihre wahrgenommene Vereinbarkeit der Pflege eines MMD mit dem Beruf beschreiben (Tab. 4).

Vorbemerkend zu erwähnen ist, dass sich – wie in der vorangegangenen Typologie – in jedem Typ Haupt- und Nebenpflegende befinden und dass diese sowohl voll- als auch teilzeitbeschäftigt sind. Niemand gibt an, die Erwerbstätigkeit aufgrund der Pflege langfristig reduziert zu haben. Auch andere Einschränkungen am Arbeitsplatz (z. B. Fehlzeiten, Karriereeinbußen) werden nicht berichtet, und niemand nutzt die Freistellungsmöglichkeiten nach Pflegezeit- und Familienpflegezeitgesetz. Dennoch nehmen alle PA Überschneidungen der beiden Lebensbereiche wahr und nutzen auf der praktischen Ebene, wenn erforderlich, Gleitzeitregelungen oder vereinzelt Urlaubstage, um der Pflege nachzukommen. Je nach Wahrnehmung der Überschneidungen nehmen sie eine bestimmte Haltung zur Vereinbarkeit von Pflege und Beruf ein. Die 3 sinngenetischen Typen (weitere Informationen im Zusatzmaterial online: Supplement 3) nennt Tab. 4.

**Table 4:** 

*Typ 1*	Getrennte Entitäten	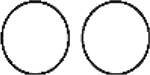
*Typ 2*	Handeln im Spannungsfeld	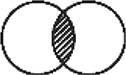
*Typ 3*	Profitable Integration	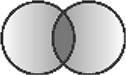

Erwerbstätige PA im Typ 1 können und möchten den Einfluss von Pflege auf den Beruf so gering wie möglich halten und lehnen hierzu Veränderungen im Berufsleben ab, z. B. den Umfang der Erwerbstätigkeit längerfristig zu reduzieren. Sie orientieren sich dabei an verschiedenen von außen bedingten oder intrinsischen Motiven (z. B. finanzielle Absicherung der Familie, Berufsidentifikation, Beruf ist Ablenkung und Erholung). Auch die Orientierung, schlichtweg nicht die Bereitschaft zu besitzen, erwerbsmäßige Anpassungen vorzunehmen (ohne andere Argumente wie z. B. die finanzielle Absicherung zu nennen), kann Motiv sein. „*Hält mich nich davon ab berufstätig zu sein […] ich hab jetzt nich das Bedürfnis zu sagen ich muss jetzt alles kündigen oder sowas und muss jetzt mich auf die Pflege meiner Mutter machen*“ (A2).

Im Typ 2 nehmen die erwerbstätigen PA Spannungen aufgrund der Überschneidung der Lebensbereiche wahr. Als Reaktion darauf passen sie die Erwerbstätigkeit an die Pflege an. Je nach familiärer Situation reduzieren oder erhöhen sie den Umfang der Erwerbstätigkeit. Eine instabile Pflege führt zur vorübergehenden Reduktion der Arbeitszeit. Zu einer Arbeitszeiterhöhung kommt es, wenn eine 24-h-Betreuungskraft angestellt werden soll, da gleichzeitig noch Kinder zu versorgen sind. Dabei nehmen sie in Kauf, dass sich die physische und psychische Belastung weiter erhöht. „*Belastend ist dieses, dass es für alle gerecht wird, dann auch noch im Beruf […] Das [Aufstocken] hat natürlich nicht zur Einfachheit beigetragen, aber ja musste halt sein*“ (F1).

Pflegende Angehörige im Typ 3 wissen um die wechselseitigen Überschneidungen und nutzen diese zum Vorteil von beiden Lebensbereichen. Sie orientieren sich dabei entweder am Einfluss der Pflege auf das Berufsleben oder umgekehrt. In der Pflege erworbene oder ausgebaute Fähigkeiten (z. B. Durchsetzungsvermögen, Organisationsgeschick) kommen dem Arbeitsplatz zugute. „*Ich glaube die Organisation, die ich durch mein Papa gelernt hab’, kommt meinem Beruf […] viel zugute*“ (C1). Das Erleben eines MMD macht sensibel und empathisch für andere Betroffene, die den PA im Arbeitskontext begegnen. Diese Entwicklung nehmen sie als positiv wahr, denn sie beinhaltet die Erfahrung, wertvoller Ansprechpartner und Zuhörer für andere Betroffene zu sein, und gibt ihnen das tröstende Gefühl, nicht allein zu sein. In 2 Fällen wirkt sich der Beruf vorteilig auf die Pflege aus. Einmal, indem die Arbeitsplatzressourcen in einem Familienbetrieb zum Erledigen von administrativen Pflegeaufgaben genutzt werden. „*Das is ja so, dass man wirklich jede Menge Sachen auch ausfüllen muss, als Angehöriger […] und das hab ich in der Firma gemacht […] also das können ja andere so gar nich*“ (E1). Zweitens, indem das arbeitsplatzbezogene Wissen eines Beratungsstellenmitarbeiters mit häuslicher Pflegeverantwortung die Organisation der Pflege erleichtert und die anderen PA im NW entlastet.

## Diskussion

Ziel dieser Pilotstudie war es, die Vereinbarkeit der informellen Pflege von MMD mit den Lebensbereichen Familie und Beruf zu ergründen. Die Ergebnisse zu den Auswirkungen der Pflege auf das Familienleben überstrahlen die Befunde zu den Wechselwirkungen mit dem Beruf. Vielmehr als Vereinbarkeitsprobleme mit dem Beruf beeinflussen familiäre Dissonanzen aufgrund der Pflege das Belastungsempfinden der PA in diesem Sample. Sie weisen auf neuartige Implikationen (z. B. therapeutische Angebote) hin, die bisher kaum eine Rolle bei der Belastungslinderung von in Deutschland lebenden PA spielten.

Die Studie zeigt den wechselseitigen Einfluss zwischen Beziehungsqualität und Aufgabenverteilung in Familien von MMD und wie sich dies auf ihre Pflegeerfahrung auswirkt. Harmonische Beziehungen und fair verteilte Aufgaben können die Pflege erleichtern, wohingegen konfliktbelastete Beziehungen und ungerecht verteilte oder ungeteilte Aufgaben für eine höhere Belastung sorgen. Die Pflege eines MMD zu Hause betrifft nicht nur Hauptpflegende, sondern auch weniger oder nichtpflegebeteiligte Verwandte, indem durch die Pflege frühere Konflikte wieder aufleben können. Die gemeinsame Pflegeerfahrung kann aber auch Chance zur positiven Entwicklung sein, indem der familiäre Zusammenhalt gestärkt wird. Diese Ergebnisse eines in Deutschland lebenden Samples werden in internationalen Studien bestätigt, wonach gute familiäre Beziehungen, geteilte Aufgaben und gegenseitige Unterstützung die Belastung reduzieren und die Gesundheit von PA verbessern [10, 27, 28]. Ebenso führen Unstimmigkeiten über die Pflege, unerfüllte Erwartungen und mangelhafte familiäre Unterstützung zu zusätzlicher Belastung [7, 19]. Dies ist insofern bedeutend, als dass PA weniger belastet und psychisch gesünder sind, wenn ihre Familien eine hohe Familienfunktionalität aufweisen [11, 29]. Mittels therapeutischer Angebote, die die Familienbeziehungen und -funktionalität von PA berücksichtigen, können positive Pflegeerfahrungen gestärkt werden [6, 31]. Doch in den hiesigen Unterstützungsangeboten für PA spielen die Beziehungen innerhalb der Familie und ihre Funktionalität bisher keine oder kaum eine Rolle. Unsere Ergebnisse sollen dazu anregen, familientherapeutische Angebote zu erwägen, die auch dem Wunsch einzelner TeilnehmerInnen entsprechen. Diese Angebote können eine innovative Ergänzung zu den bestehenden formellen und informellen Unterstützungsangeboten sein, die – so auch in unserem Sample – essenziell zum Erbringen der Pflege und zur Entlastung der PA sind.

Die Studie zeigt auch, dass erwerbstätige PA von MMD die Lebensbereiche Pflege und Beruf als getrennt voneinander, miteinander in Konflikt geratend oder sich gegenseitig unterstützend wahrnehmen können. Unseres Wissens nach beschreiben diese Ergebnisse erstmalig unterschiedliche Betrachtungsweisen der Betroffenen zur Vereinbarkeit von Pflege und Beruf. Zudem zeigen die Interviews auf einer praktischen Ebene Bedingungen auf, die die Vereinbarkeit der Pflege mit dem Beruf begünstigen. Hierzu zählen eine pflegesensible Unternehmenskultur, die u. a. eine flexible Arbeitszeitplanung erlaubt, ein von vornherein reduzierter Beschäftigungsumfang und stabilisierende Kontextfaktoren wie die NW-Zusammensetzung aus familialen und externen Personen oder Leistungsanbietern. Diese Bedingungen sind – neben anderen beeinflussenden Faktoren – bereits gut untersucht [2, 16, 17, 23].

Als Schwäche der Studie ist ein Selektionsbias zu nennen, wofür qualitative Studien grundsätzlich anfällig sind. Die Rekrutierung der TeilnehmerInnen gelang nur durch persönliche Vermittlung der Gatekeeper. Auch TeilnehmerInnen schränkten die Sample-Zusammensetzung ein, indem es ihren Aussagen nach nicht möglich gewesen sei, Verwandte, die sich beispielsweise weitgehend aus der Pflege zurückhielten, zur Studienteilnahme zu gewinnen. Die begrenzte Stichprobengröße dieser explorativen Studie schließt das nachträgliche Rekrutieren kontrastierender Fälle aus (z. B. PA, die ihre Arbeitszeit aufgrund der Pflege langfristig reduzierten, oder PA mit anderen Verteilungen hinsichtlich der soziodemografischen Merkmale).

Die Stärke der Studie liegt in dem rekonstruktiven Ansatz, der neue Erkenntnisse zur erlebten Vereinbarkeit von informeller Pflege, Familie und Beruf liefert. Diese wurden zudem erstmals auf Basis einer NW-Perspektive erhoben, indem durch die Befragung von Haupt- und Nebenpflegenden verschiedene NW-Konstellationen abgebildet wurden [4] und diese mittels komparativer Sequenzanalyse, methodisch kontrolliert, in der Interpretation berücksichtigt wurden [20]. Die Interpretation des umfassenden Textmaterials erfolgte durch Diskurs im Team und mit anderen ForscherInnen. Die Ergebnisse bilden einen Ausgangspunkt für weitere Forschung, die beispielsweise prüft, ob sich die Typologien möglicherweise auch in anderen Stichproben aus PA wiederfinden, oder die den qualitativ ergründeten Zusammenhang zwischen Beziehungsqualität, familiärer Organisation der Pflege und Belastungsempfinden von PA mittels quantitativer Designs untersucht.

## Fazit für die Praxis

- Die Pflege eines Menschen mit Demenz (MMD) belastet das Familien- und Berufsleben von pflegenden Angehörigen (PA), was sich wiederum nachteilig auf ihre Gesundheit auswirken kann. Doch Familie und Beruf können auch als Puffer fungieren und pflegebedingte Belastung reduzieren.
- PA brauchen das Wissen über bestehende Versorgungsangebote, um sich selbst und ihre Familie durch ein verlässliches Netzwerk aus formellen Dienstleistern und informellen Personen (z. B. Ehrenamtliche) zu unterstützen.
- Zu Stressbewältigung und Stärkung positiver Pflegeerfahrungen können zudem therapeutische Angebote hilfreich sein, die auch die Familienbeziehungen und -funktionalität von PA berücksichtigen.
- Der Beruf kann erwerbstätigen PA Erholung bieten und ihr Wohlbefinden fördern. Um dem Wunsch vieler PA, trotz Pflege berufstätig zu bleiben, entgegenzukommen, werden PA auch in Zukunft eine nicht zu vernachlässigende Zielgruppe bei Maßnahmen zur besseren Vereinbarkeit von Familie und Beruf sein.

## Caption Electronic Supplementary Material






