# Supplementary material for: Compatibility of caring in dementia, family and employment
Source: Z Gerontol Geriatr. 2020 Jul 27;54(7):643–50. [Article in German] doi: 10.1007/s00391-020-01764-9 (PMC8551114; doi:10.1007/s00391-020-01764-9)
Supplement: Supplementary file 1 [file 391_2020_1764_MOESM1_ESM.pdf]

Ergänzendes Online-Material 1:

Dieses Dokument liefert vertiefende Informationen zum Design der *mixed methods* Pilotstudie mit Fokus darauf, wie die Netzwerkperspektive in der Interpretation der Interviews Berücksichtigung findet. Abbildung 1 stellt das Studiendesign im Überblick dar.

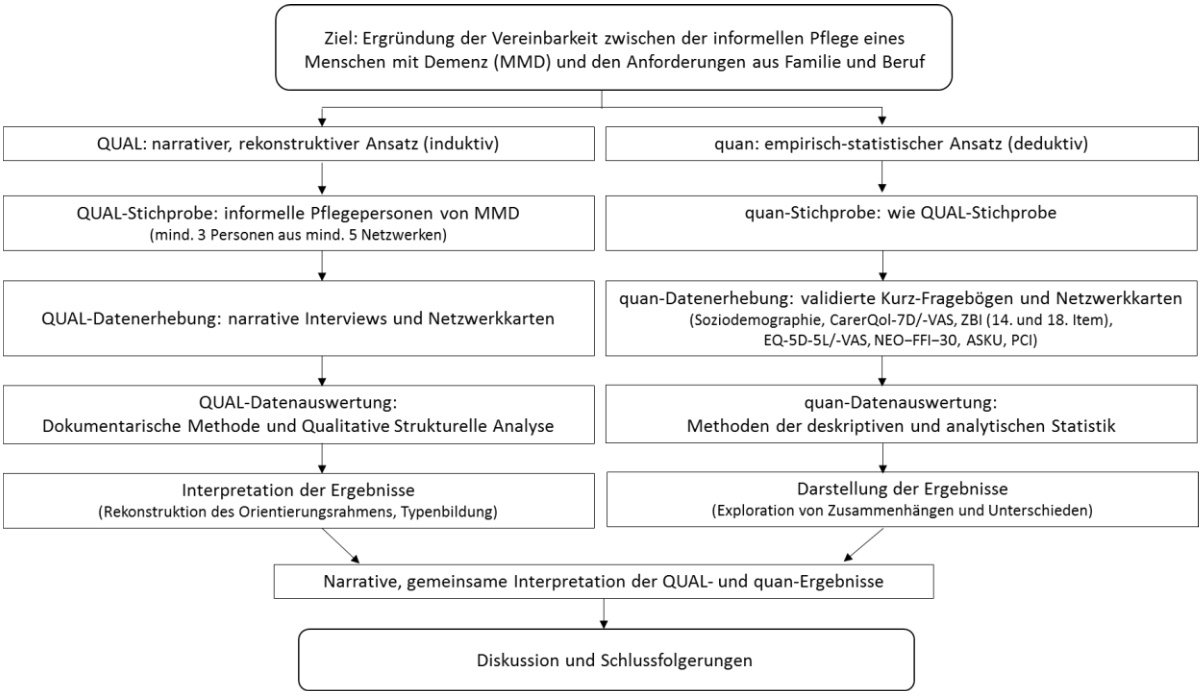

Abbildung 1: Abbildung des Studiendesigns. Simultaneous Qualitatively Driven Mixed Methods Design (QUAL+quan Design); MMD, Menschen mit Demenz; CarerQol-7D; CarerQol-VAS (Deutsche Version); ZBI, Zarit Burden Interview (Deutsche Version); EQ-5D, EQ-VAS (Deutsche Version); NEO-FFI-30, Neo-Fünf-Faktoren Inventar-30 (Deutsche Version); ASKU, Allgemeine Selbstwirksamkeitsskala; PCI, Proaktives Coping Inventar (Deutsche Version)

Die Studie wurde als *simultaneous qualitatively driven mixed methods design* (*QUAL+quan design*) konzipiert. Das heißt, beide Forschungsstränge sollen sich ergänzen, wenn auch der qualitative Forschungsstrang priorisiert wird. Im QUAL-Teil wurden narrative Interviews [6] und zu deren Auswertung die Dokumentarische Methode [5] eingesetzt. Im quan-Teil wurden standardisierte Fragebögen genutzt, die – angesichts der kleinen Stichprobe – deskriptiv ausgewertet wurden. Eine Zwischenposition nehmen **Netzwerkkarten** nach der Methode von Kahn and Antonucci [4] ein, die die StudienteilnehmerInnen im Anschluss an die Interviews ausgefüllt haben. Diese spiegeln die Struktur des jeweiligen Netzwerks (Akteure und Verbindungen unter ihnen) wider. Zur ausführlichen Beschreibung der Erhebungsinstrumente (narrative Interviews, Fragebögen) und ihre Auswertungsmethoden (Dokumentarische Methode, deskriptive und analytische Statistik) sei auf das *open access*-veröffentlichte Studienprotokoll [1] verwiesen.

Anhand eines konkreten Beispiels soll nun das Vorgehen beschrieben werden, wie die **Netzwerkperspektive** in der Interpretation berücksichtigt wurde. An der Studie nahm ein Netzwerk aus drei Geschwistern teil, die sich gemeinsam um ihre an Demenz erkrankte Mutter kümmern. Jedes Geschwister füllte eine Netzwerkkarte aus. Von Studienteilnehmerin A-3 sah diese so aus (Abbildung 2):

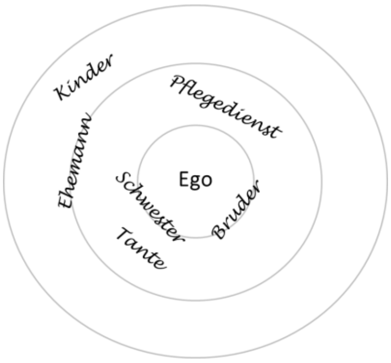

Abbildung 2: Beispiel einer egozentrierten Netzwerkkarte (Studienteilnehmerin A-3)

Im nächsten Schritt wurden die einzelnen Netzwerkkarten von den befragten Netzwerkmitgliedern kombiniert, so dass eine gemeinsame Netzwerkkarte für dieses Familiennetzwerk, Netzwerk A, vorlag (Abbildung 3).

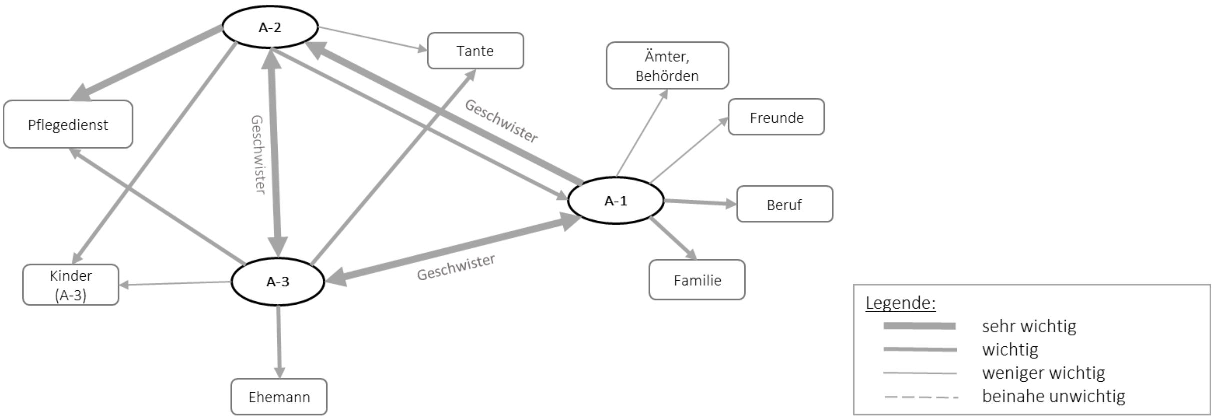

Abbildung 3: Kombinierte Netzwerkkarte (Netzwerk A; relationaler Typ 1)

Die kombinierten Netzwerkkarten wurden anhand des von Herz et al. [3] vorgeschlagenen Vorgehens interpretiert. Das heißt, wir haben Fragen an die Netzwerkkarten gestellt, zu denen wir dann nach Antworten im qualitativ erhobenen Material aus den Interviews gesucht haben. Ebenso offenbarten die Netzwerkkarten Zusammenhänge bzw. Verbindungen, die in den Interviews nicht zur Sprache kamen. Auf diese Weise findet die Netzwerkperspektive in den interpretierten Interviews Berücksichtigung. Nach Interpretation der einzelnen Interviews mittels Dokumentarischer Methode lässt sich für das betrachtete Netzwerk A festhalten:
Netzwerk A (relationaler Typ 1) besteht aus drei Geschwistern, die die Pflege als ein gemeinsames Projekt wahrnehmen. Jedes von ihnen hat seine Betreuungsaufgaben. Die Geschwister selbst bilden die Basis des Netzwerks und nennen nur wenige externe Unterstützungsressourcen. Diejenigen, die für sie wesentlich sind, überschneiden sich (z.B. Pflegedienst), insbesondere zwischen A-2 und A-3. A-1 scheint etwas isolierter zu sein. Diese Interpretation wird durch die qualitativen Befunde gestützt, bei denen A-2 und A-3 stärker in die praktische Pflege eingebunden sind und die primäre Rolle von A-1 die Verwaltung der Pflege ist.

Die kombinierten Netzwerkkarten zeigen, dass diese Form der Visualisierungen durch die Erzählungen unterstrichen wird und mit den Ergebnissen der Typenbildung verknüpft ist. In zwei

Netzwerken repräsentieren die Netzwerkmitglieder den gleichen Typ. In den übrigen fünf Netzwerken ist dies nicht der Fall, da die Typenbildung auf Basis der einzelnen Interviews mittels Dokumentarischer Methode (sprich auf Individualebene) durchgeführt wurde (hierzu siehe auch [2]).

1. Blinded (2018)
2. Blinded (2020)
3. Herz A, Peters L, Truschkat I (2014) How to do Qualitative Structural Analysis: The Qualitative Interpretation of Network Maps and Narrative Interviews. Forum Qualitative Sozialforschung/Forum Qualitative Social Research 16
4. Kahn RL, Antonucci TC (1980) Convoys over the life course: Attachment, roles, and social support. Academic Press, New York
5. Nohl A-M (2017) Interview und Dokumentarische Methode - Anleitungen für die Forschungspraxis. Springer VS, Wiesbaden
6. Schütze F (1976) Zur Hervorlockung und Analyse von Erzählungen thematisch relevanter Geschichten im Rahmen soziologischer Feldforschung: Dargestellt an einem Projekt zur Erforschung von kommunalen Machtstrukturen. In: Weymann A (Hrsg) Kommunikative Sozialforschung: Alltagswissen und Alltagshandeln, Gemeindemachtforschung, Polizei, politische Erwachsenenbildung. Fink, München S159-260
