# Supplementary material for: Compatibility of caring in dementia, family and employment
Source: Z Gerontol Geriatr. 2020 Jul 27;54(7):643–50. [Article in German] doi: 10.1007/s00391-020-01764-9 (PMC8551114; doi:10.1007/s00391-020-01764-9)
Supplement: Supplementary file 2 [file 391_2020_1764_MOESM2_ESM.pdf]

Ergänzendes Online-Material 2:

Beschreibung der fünf Typen (T) pflegender Angehöriger von Menschen mit Demenz hinsichtlich der wahrgenommenen Vereinbarkeit von Pflege und Familie. Die relationalen Typen wurden auf Grundlage des wechselseitigen Einflusses zwischen Beziehungsqualität und Aufgabenverteilung innerhalb der Familie rekonstruiert.

	**Beschreibung des Typs**<br>(MMD=Mensch mit Demenz)	**Zitate**<br>(*Auslassungen und Erläuterungen stehen in []*)
**T1: Gemeinschafts-projekt**<br><br>*(wir pflegen gemeinsam)*	Dieser Typus zeichnet sich durch Einigkeit und Harmonie zwischen den pflegenden Familienmitgliedern aus. Sie können sich gegenseitig aufeinander verlassen, sie besprechen zu fällende Entscheidungen gemeinsam und finden Lösungen einvernehmlich.<br><br>Sie teilen gemeinsame Wertvorstellungen, wie z.B. die Verantwortung für den MMD zu übernehmen und sehen dies als ihre Kindespflicht den Eltern gegenüber an. Ebenso ist es ihnen wichtig, dass jeder von ihnen darauf achtet, für sich selbst gut zu sorgen. Die Aufgabenverteilung muss nicht ausgehandelt werden, sondern ergibt sich wie selbstverständlich auf Grundlage individueller Fähigkeiten, charakterlicher Eigenschaften und traditioneller Geschlechterrollenbilder.<br><br>Die Familienmitglieder dieses Typs stehen sich persönlich sehr nahe. Die geteilte Pflegeverantwortung hat den Kontakt zwischen den pflegenden Angehörigen und deren Familien sogar intensiviert.<br><br>Diese Eigenschaften bilden die Basis dafür, dass die Pflegeaufgaben auf den Schultern der pflegenden Angehörigen verteilt sind. Auch wenn quantitativ gesehen ein Familienmitglied die meiste Zeit mit dem MMD verbringt, wird die Pflege als Gemeinschaftsprojekt verstanden, zu dem jede(r) seinen Beitrag leistet. Die geteilte Pflegeverantwortung und das harmonische Verhältnis zwischen den Familienmitgliedern sorgen dafür, dass die häusliche Pflegesituation nahezu als leicht zu bewerkstelligen wahrgenommen wird. Sollten sich die Bedingungen zuhause verändern, so wird einer Anpassung des Pflegearrangements zuversichtlich entgegengeblickt.	*<Das is halt auch das Schöne dass ich mich da auch drauf verlassen kann [auf die Beteiligung der Geschwister] man hat immer so man weiß ’Ach der Andere ist ja auch noch da und macht das’ ((Luftholen)) oder kommt dann wenn man dann wirklich absolut mal gar kein äh Zeit hat oder keine Lust> (Hauptpflegende, Netzwerk A)*<br><br>*<Das war eigentlich n Selbstgänger das hat sich eigentlich ohne große Absprachen so ergeben, typbedingt auch […] und ich denk mir mal meine Mutter würde es auch nie wollen dass mein Bruder sie wäscht. Das ist Tradition noch und ich glaub er würde es auch nicht schön finden> (Nebenpflegende, Netzwerk A)*<br><br>*<Wir hatten schon immer ein gutes Verhältnis, […] jetzt mit der Pflege ist es sowieso dass wir uns mehr oder weniger ständig austauschen […] und der Kontakt untereinander is mehr geworden und intensiver geworden […] die Pflege ist der Aufhänger aber schon mit dem zweiten dritten Satz ist dann ’Wie geht es deiner Tochter? Treffen wir uns zum nächsten Geburtstag?’> (Nebenpflegender, Netzwerk A)*

**T2: Kooperation mit externer Hilfe**<br><br>*(wir haben ein Back-up)*	Auch für diesen Typ ist es selbstverständlich, sich um den MMD zu kümmern und dessen Wohlergehen in den eigenen vier Wänden zu sichern. Die Besonderheit hier ist, dass weitere Familienangehörige, die potenziell unterstützend tätig sein könnten, nicht verfügbar sind oder von ihnen keine Beteiligung erwartet wird. Der solitär pflegende Angehörige ist mit seiner familiären Alleinstellung einverstanden, keiner der betrachteten Fälle beklagt sich darüber oder nimmt pflegebedingte Auswirkungen auf die Beziehung zu weiteren Verwandten (wenn vorhanden) wahr.<br><br>Die Bedeutung externer Dienste zur Sicherstellung der häuslichen Pflege ist in diesem Typ besonders ausgeprägt. Zusätzlich zu ambulanten Pflegediensten lässt sich die Pflege nur mit Hilfe von ehrenamtlichen oder professionellen Betreuungskräften mit persönlichen Interessen oder einer Erwerbstätigkeit vereinbaren. Ist die Betreuung nicht sichergestellt, müssen Freizeitaktivitäten oder auch in einem Fall die Berufstätigkeit zeitweise zurückstecken.<br><br>Bis ein externes Unterstützungsnetzwerk aufgebaut ist, wird der Alltag als überlastet und von Sorgen geprägt wahrgenommen. Voraussetzung für die Kooperation mit nicht-familialen Betreuungskräften ist, dass die Pflege als plan- und organisierbar erlebt wird und sich die Angehörigen auf die Ersatzpersonen verlassen können. Als befreiend wird es wahrgenommen, wenn dann Freizeitvergnügen oder die Berufstätigkeit wieder aufleben können.	*<Letzte Woche war ich zum Beispiel am Donnerstag und Freitag in [Stadt in Deutschland] zu einem Kursus; das hätte ich mir auch nicht erlauben können wenn wir nich=n Back-up gehabt hätten> (Solitär Pflegender, Netzwerk D)*<br><br>*<Dann ja doch ne ganze Menge kommt was macht man was tut man wo geht man hin wo kriegt man Hilfe kriegt man überhaupt Hilfe, […] und da war=s dann wahrscheinlich schon so eben deswegen auch dass die Nerven mal zusammengefallen waren dass ich dann nur noch ((klopfendes Geräusch)) das Wasser aus den Augen lief. Aber nö jetzt […] bei der Betreuung die hat=s im Griff; wenn denn irgendwas is dann meldet sie sich und denn is gut> (Solitär Pflegender, Netzwerk G)*<br><br>*<Ich wusste nicht was ich überhaupt unternehmen kann oder darf ohne permanent ein schlechtes Gewissen zu haben. Wenn irgendwo n RTW fuhr mit Blaulicht und Sirene sträubten sich mir die Nackenhaare und das is schon ne Belastung gewesen und jetzt mit der [Betreuungskraft] hab ich da äh den Kopf frei der Nacken entspannt sich> (Solitär Pflegender, Netzwerk D)*
**T3: Kooperation in der Familie**<br><br>*(wir funktionieren)*	Gemeinsam ist den Individuen dieses Typs die Orientierung, dass es ihrem Familienverständnis entspricht, einen verwandten MMD zuhause zu pflegen. Wenngleich die Aufgaben ungleichmäßig verteilt sind, so wird diese Verteilungsdysbalance akzeptiert. Hauptpflegende dieses Typs erwarten keine intensivere familiäre Beteiligung und Nebenpflegende sehen ihre Möglichkeiten ausgeschöpft (z.B. aufgrund von Schulabschluss, Berufstätigkeit, weite Entfernung). Für die Hauptpflegenden lässt sich das Vertrauen rekonstruieren, sich auf (mehr) familiäre Unterstützung verlassen zu können, falls sie erforderlich sein sollte. Auch können sie darauf vertrauen, dass sie gemeinsam eine Lösung finden, wenn sich die Pflegesituation verändern sollte.	*<Wir haben kein so=n enges Verhältnis dass wir uns also so regelmäßig seh=n aber ich weiß dass ich mich hundert Prozent auf ihn verlassen kann> (Hauptpflegende, Netzwerk C)*<br><br>*< Natürlich könnt ich mit meiner Mutter jetzt nich unbedingt in Urlaub fahr=n. Das is klar. Aber ich glaube dass sich selbst dafür ne Lösung finden würde [...] dass mein Onkel irgendwie dann paar Tage hier is oder sonst was also ne Lösung würde sich immer finden> (Nebenpflegende, Netzwerk C)*

	Die Angehörigen zollen sich gegenseitige Anerkennung – unabhängig davon wie groß der jeweilige Beitrag zur Pflege ist. Die Verbindung zwischen Aufgabenverteilung und Beziehungsqualität innerhalb der Familie gestaltet sich in diesem Typ ohne Reibungen, d.h. die ungleich verteilte Pflege des MMD belastet die Beziehungen nicht und die bestehenden Beziehungen enthalten kein Konfliktpotenzial für das ungleich verteilte Ausgestalten der Pflege.<br><br>Die Angehörigen sind mit ihrem momentanen, ungleich verteilten Pflegearrangement einverstanden, es ist für sie kooperativ und es funktioniert. Gleichwohl nehmen Nebenpflegende wahr, dass die Aufgabenverteilung nicht gerecht ist und zu Lasten der Hauptpflegenden geht – was wiederum das familiäre Klima bedroht.	*<Die Mama in der Hinsicht halt dass sie so viel abgenomm hat; das is halt schon eine ähm Erleichterung für uns alle gewesen; aber man hat es halt dann wiederum bei ihr gemerkt weil=sie sehr gestresst war was sich wiederum nich ganz so positiv auf uns ausgelöst also ausgewirkt hat> (Nebenpflegende, Netzwerk F)*
**T4: Enttäuschung**<br><br>*(wir sind enttäuscht)*	Ein nicht erfülltes Idealbild von familiärer Unterstützung und starke Enttäuschung darüber bestimmen diesen Typ. Informell Pflegende dieses Typs pflegen aus Dankbarkeit, Liebe und moralischer Verpflichtung dem MMD gegenüber. Es ist für sie selbstverständlich. Dies erwarten sie auch von ihren Verwandten, die aber diesem Ideal nicht nachkommen. Die Pflegenden fühlen sich allein gelassen, ausgenutzt, unverstanden und sind sehr verletzt.<br><br>Als Kontrast in der Gemeinsamkeit dieses Typs zeigen sich unterschiedliche Umgangsweisen mit der enttäuschten Erwartung. Wenngleich alle an ihrem Ideal von familiärer Unterstützung festhalten, so zeigt sich eine Ausprägung, in der um den Beitrag für die Pflege des MMD von den anderen Familienmitgliedern gekämpft wird. Individuen einer weiteren Ausprägung hingegen versuchen nicht, andere Familienmitglieder zur Unterstützung zu bewegen, sondern scheuen Konflikte mit und Abhängigkeiten von ihnen. Stattdessen werden diese durch größere Einspannung externer Dienste substituiert.<br><br>Wiederum gemeinsam ist diesem Typ, dass es aufgrund der ungleich verteilten Pflegeverantwortung und der Enttäuschung darüber zum Beziehungsabbruch zu den Familienmitgliedern kommt. Unabhängig davon ob die Pflegenden noch um deren Beteiligung an der Pflege kämpfen oder die Hoffnung darauf bereits aufgegeben haben, sie kündigen ihnen die Beziehung in anderen Bereichen. Das	*<Dass einfach dann gesagt wird 'Da der eine hier is im Haus und der soll gefälligst auch gucken wie er klar kommt' ne; das is zwar hart also für mich ganz hart weil ich vom äh von meinem Verständnis her einfach das nicht äh in die Reihe krieg> (Nebenpflegender, Netzwerk F)*<br><br>*<Ich werde nich aufgeben ((Luftholen)) bevor die [Geschwister] nich äh anfangen zumindest n Stück weit ihre Rolle zu übernehmen die ich ihnen schon zugedenke in so einer Situation; ((Luftholen)) ähm so langsam werden sie tatsächlich wach aber ich bin seit 2 Jahren dabei> (Hauptpflegende, Netzwerk E)*<br><br>*<Ich hab keine Familie mehr weil die unterstützt mich kein Stück mit meiner Mutter ich sag 'Echt lasst mich in Frieden' […] aus 'Gemeinsam sind wir stark' is geworden 'Ich hab so wenig Kontakt wie möglich zu ihnen'> (Hauptpflegende, Netzwerk E)*

	Zusammenkommen mit der Familie, sich in anderen Bereichen auszutauschen oder zu unterstützen, wird gemieden oder gar verweigert (bspw. indem Familienfeste getrennt voneinander verbracht werden oder nachgefragte Hilfe zu anderen familiären Themen abgelehnt wird).	
**T5: Zwangssituation**<br><br>*(wir haben keine Alternative)*	Den Pflegenden in Typ 5 ist gemeinsam, dass sie ihre Situation als nicht zufriedenstellend wahrnehmen, diesen Zustand aber aufgrund von Alternativlosigkeit aushalten (müssen). Wie die vorangegangen Typen pflegen sie aus moralisch-gesellschaftlicher Verpflichtung, aus Liebe und Dank sowie zum Wohl des MMD. Auch diesen Fällen ist ihre Orientierung an familiärem Zusammenhalt gemeinsam. Doch die Aufgaben im Rahmen der Pflege sind ungleich verteilt und die Vorstellungen von Art und Umfang gegenseitiger Unterstützung differieren zwischen den Familienmitgliedern. Daraus resultieren Konflikte auf der Beziehungsebene, für die nur kurzfristige Lösungen gefunden werden oder Lösungen, in der schließlich eine Partei um des Friedens willen nachgibt. Nicht nur die Pflegesituation belastet die Beziehungen, sondern die Fälle offenbaren, dass es bereits in Zeiten vor der Pflege Spannungen gab (z.B. aufgrund eines schwierigen Verhältnisses zwischen der Kern- und Herkunftsfamilie).<br><br>Kontraste sind zu erkennen, je nachdem ob es sich um Haupt- oder Nebenpflegende handelt. Erstere empfinden ihre Rolle als aufgezwungen und beklagen die Vernachlässigung anderer Lebensbereiche und persönlicher Bedürfnisse aufgrund der permanenten Präsenz, die von ihnen physisch wie mental für die Bewerkstelligung der Pflege gefordert wird. Wohnen gleichzeitig noch schulpflichtige Kinder im Haus, leiden sie unter der Wahrnehmung, diesen nicht mehr gerecht zu werden. Auch wenn sie sich auf der einen Seite gezwungen fühlen, die Hauptlast zu tragen, widerspricht es andererseits ihren Werten diese aufzugeben. Nebenpflegende halten daran fest, sich (aus beruflichen und familiären Gründen) nicht mehr einbringen zu können. Auch wenn es sie teilweise beunruhigt, zu sehen, wie die Hauptpflegeperson die ungeliebte Rolle ausführt, zollen sie ihr Anerkennung und Wertschätzung, um sie zur Pflege zu motivieren. Sie bringen sich im Rahmen ihrer begrenzten Möglichkeiten ein, doch eine Umgestaltung der Pflege, die auf beiden Seiten für langfristige Zufriedenheit sorgen würde, ist auch ihnen nicht möglich.	*<Ich war dann eigentlich diejenige die die entschuldigen Sie Arschkarte hatte ähm und [Name der Schwester] kam immer nur vorbei und 'Atütütü und atatata und willst du das nicht so machen, und willst du das nicht so machen' ((Luftholen)) und ich hatte hier ein Tach mir stand das Wasser Oberlippe und Oberkante Unterlippe und meine Schwester kam mit klugen Vorschlägen und hatte von nichts ne Ahnung> (Hauptpflegende, Netzwerk B)*<br><br>*<Die Spontanität die weg war die hat es mir ganz arg schwer gemacht. Ich weiß am ersten Mai ((Luftholen)) bin ich mit dem Rollstuhl und der Schwiegermutter […] heimgelaufen und hab dann wirklich die letzten paar Meter geheult weil ich dachte 'So und die anderen Familien gehen jetzt mit ihren Kindern Fahrradfahren oder sonst irgendwas' und […] ich hab jetzt die Schwiegermutter an der Backe und kann nicht mal spontan fort. Und ich hab immer so das Gefühl gehabt es geht an den Kindern ab […] da hab ich immer n=schlechtes Gewissen einfach auch gehabt> (Hauptpflegende, Netzwerk F)*<br><br>*<Sie hat ja dann hier wirklich den Hauptstress und sie hat ja hier wirklich großen Einsatz gezeigt das muss man auch zeigen und das=sie damit jetzt nicht mehr fertig wird kann ich auch gut verstehen […] ich seh natürlich was sie hier leistet und was sie hier tut […] das seh ich alles und von daher hab ich=s vielleicht in manchen Dingen auch wirklich besser trotz meiner bestimmt nicht weniger Arbeit*

	Individuen dieses Typs halten aus Mangel an Alternativen an dem momentanen Pflegearrangement fest, welches auf einem familienbiographischen Boden gewachsen ist.	*[Berufstätigkeit]. Aber äh also ich möcht nich mit ihr tauschen muss ich ganz ehrlich sagen insofern find=ich das auch toll wie sie das hier macht aber es stößt uns eben halt so die Art des Umgangs und das stößt uns ab und zu wirklich erheblich auf das muss man schon so sagen> (Nebenpflegende, Netzwerk B)*
