# Supplementary material for: Compatibility of caring in dementia, family and employment
Source: Z Gerontol Geriatr. 2020 Jul 27;54(7):643–50. [Article in German] doi: 10.1007/s00391-020-01764-9 (PMC8551114; doi:10.1007/s00391-020-01764-9)
Supplement: Supplementary file 3 [file 391_2020_1764_MOESM3_ESM.pdf]

Ergänzendes Online-Material 3:

Beschreibung der drei Typen (T) pflegender Angehöriger von Menschen mit Demenz hinsichtlich der wahrgenommenen Vereinbarkeit von Pflege und Beruf. In diesen sinngenetischen Typen wurden typisierte Haltungsorientierungen von erwerbstätigen PA rekonstruiert, die ihre wahrgenommene Vereinbarkeit beider Lebensbereiche beschreiben.

	**Beschreibung des Typs**<br>(MMD=Mensch mit Demenz)	**Zitate**<br>(*Auslassungen und Erläuterungen stehen in []*)
**T1: Getrennte Entitäten**<br>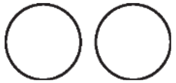	Erwerbstätige, pflegende Angehörige dieses Typs können und möchten die Beeinflussung der Pflegeverantwortung auf den Beruf so gering wie möglich halten. Sie lehnen Veränderungen im Berufsleben, wie z.B. den Umfang der Erwerbstätigkeit zu Gunsten der Pflege längerfristig zu reduzieren, ab. Dafür orientieren sie sich an verschiedenen Motiven, die entweder durch äußere Gegebenheiten oder intrinsisch bedingt sind.<br><br>Der Beruf ist die finanzielle Absicherung des eigenen und des familiären Lebensunterhalts (insbesondere, wenn die pflegenden Angehörigen Kinder haben, die sie noch versorgen oder zumindest finanziell unterstützen). Auch ist der Beruf ein wichtiger Bestandteil der eigenen Persönlichkeit und trägt zu deren Verwirklichung bei. Angehörigen dieses Typs ist es wichtig, ihre Karriere weiterzuverfolgen oder ihr Ansehen im Beruf zu erhalten.<br><br>Eine weitere Orientierung dafür, in der Erwerbstätigkeit keine Einschränkungen erfahren zu wollen, ist das Wahrnehmen des Berufs als Ablenkung zur und Erholung von der Pflegetätigkeit. Auch die Orientierung, schlichtweg nicht die Bereitschaft zu besitzen, erwerbsmäßige Anpassungen vorzunehmen (ohne andere Argumente wie z.B. die finanzielle Absicherung zu nennen), kann Motiv sein.<br><br>Zuletzt unterstützt der Kontextfaktor, weit entfernt vom zu pflegenden MMD zu wohnen, die Haltung, nach der die gegebenen Umstände keine weitere Vereinbarkeit von Pflege und Beruf zulassen.	*<Ich bin gut zwölf dreizehn zwölf Stunden weg […] fahr dann häufig hier nochmal abends vorbei […] ich=hab auch immer einfach so das Gefühl ich muss noch mal kurz hier rein gucken […] aber ich hab dann natürlich auch irgendwann das Bedürfnis dass ich mal (.) mal nach Hause möchte und essen möchte und sowas> (Nebenpflegende, Netzwerk B)*<br><br>*<Da aber eben auch halt auch auf andere Gedanken kommt und nich immer nur an die Krankheit und an die Probleme zuhause dann denkt […] wenn man da einmal raus is und der hat n=Andern genomm' denn kommt man auch kaum wieder rein und denn fehlt mir ja das Geld […] ich kann=s mir nich leisten dort jetzt einfach zuhause zu bleiben> (Solitär Pflegender, Netzwerk G)*<br><br>*<[Die Pflege] hält mich nich davon ab berufstätig zu sein jetzt oder sozusagen also ich hab jetzt nich das Bedürfnis zu sagen ich muss jetzt alles: äh kündigen oder sowas und muss jetzt mich auf die Pflege meiner Mutter machen> (Nebenpflegende, Netzwerk A)*

**T2: Handeln im Spannungsfeld**<br>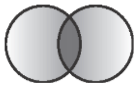	Im Gegensatz zum ersten Typ lässt sich bei Angehörigen des zweiten Typs die Wahrnehmung rekonstruieren, dass sich die Verantwortlichkeiten aus Pflege und Beruf überschneiden und in Konflikt geraten können. Dieses Spannungsfeld erfordert eine Reaktion von ihnen, mit der die Angehörigen dieses Samples die Erwerbstätigkeit an die Pflege anpassen. Ihre Reaktion fällt je nach familiärer (Ausgangs-) Situation in beide denkbare Richtungen aus: sie reduzieren oder erhöhen den Umfang der Erwerbstätigkeit.<br><br>Zur Reduktion der Arbeitsstunden kommt es, wenn bspw. die Pflegesituation des MMD zuhause als instabil wahrgenommen und ihre Lösung in der (zeitweise) eingeschränkten Erwerbstätigkeit gesehen wird. Die Arbeitsstunden werden wieder erhöht, sobald die Stabilität wiederhergestellt ist. Und auch wenn der Fall noch nicht eingetreten ist, erwägen Angehörige dieses Typs zumindest gedanklich eine mögliche, künftige Arbeitszeitreduktion zu Gunsten der Pflege.<br><br>Zu einem höheren Umfang der Erwerbstätigkeit (Aufstocken der Arbeitszeit oder Aufnahme einer zusätzlichen Beschäftigung) kommt es, wenn der Beruf nicht nur den familiären Lebensunterhalt sichert, sondern auch die Pflege zuhause durch die zusätzliche Inanspruchnahme einer 24 Stunden-Betreuung gewährleistet werden soll. Dass sich durch das Aufstocken der Arbeitszeit die physische und psychische Belastung erhöht, wird von den Betroffenen in Kauf genommen.	*<Ich hab also zwei Standbeine zwei kleine Firmen […] und die zweite Firma […] ist mit einer Reisetätigkeit verbunden in der Regel weil ich da bundesweit unterwegs war ((Luftholen)) das is natürlich völlig eingebrochen die letzten zwei Jahre und jetzt wieder möglich> (Solitär Pflegender, Netzwerk D)*<br><br>*<Das sind so Ideen die ich hab wenn sie immer noch in dem Haus wohnt […] wenn das noch einigermaßen geht ob ich das [die Arbeitszeit zu reduzieren] dann wirklich machen würde […] ich weiß auch nich […] n=bisschen arbeiten wollt ich schon auch noch> (Hauptpflegende, Netzwerk A)*<br><br>*<Belastend ist dieses dass es für alle gerecht wird dann auch noch im Beruf […] Das [Aufstocken] hat natürlich nicht zur Einfachheit beigetragen aber ja musste halt sein. Gibt manchmal Gründe dass man sich so entscheidet> (Hauptpflegende, Netzwerk F)*
**T3: Profitable Integration**<br>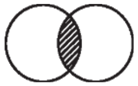	Angehörige des dritten Typs wissen um die Wechselwirkungen zwischen den Lebensbereichen Pflege und Beruf und können diese – als Unterschied zum zweiten Typ – für beide Verantwortungsbereiche vorteilhaft nutzen. Sie orientieren sich dabei entweder am Einfluss der Pflege auf den Beruf oder umgekehrt am Einfluss des Berufs auf die Pflege. Ist letzteres der Fall, so liegt dies an spezifischen Besonderheiten der jeweiligen hier untersuchten Fälle.<br><br>In der Pflegetätigkeit erworbene oder ausgebaute Fähigkeiten können auch dem Arbeitsplatz zu Gute kommen. Insbesondere wenn die Pflege eines MMD von mehreren externen Diensten unterstützt wird, erfordert dies Planungs- und Organisationsgeschick von den pflegenden Angehörigen. Ebenso kann ein gutes	*<Ich glaube die Organisation die ich durch mein Papa gelernt hab kommt zu=meinem Beruf […] und jetzt hier privat äh viel zugute> (Hauptpflegende, Netzwerk C)*<br><br>*<Also das is ja so dass man wirklich jede Menge Sachen auch ausfüllen muss als Angehöriger ((Luftholen)) und auch natürlich für meine Mutter so regeln muss und da hab ich so gedacht ‘das machst du nich zuhause’ und=das hab ich in=der Firma gemacht; […] also das könn=ja andere so gar nich> (Hauptpflegende, Netzwerk E)*

	Durchsetzungsvermögen von Vorteil sein – in der Pflege genauso wie am Arbeitsplatz. Das persönliche Erleben eines MMD in der Familie macht sensibel und empathisch für andere Betroffene, die den Angehörigen dieses Samples u.a. im Arbeitskontext begegnen. Diese Entwicklung nehmen sie als positiv wahr, denn sie beinhaltet nicht nur die Erfahrung, wertvoller Ansprechpartner und Zuhörer für andere Betroffene sein zu können, sondern auch das tröstende Gefühl mit den eigenen Problemen nicht allein zu sein.<br><br>In zwei Fällen wirkt sich die Erwerbstätigkeit vorteilig auf die Pflege aus. Eine (haupt-) pflegende Angehörige ist in der Firma ihres Bruders tätig und sie nutzt die Arbeitszeit dort, um administrative Pflegeaufgaben zu erledigen. Ein anderer Angehöriger dieses Samples ist in einer Beratungsstelle für hilfe- und pflegebedürftige Menschen und deren Angehörige beschäftigt. Dadurch kann er die administrativen Rahmenbedingungen der häuslichen Pflege ohne großen Aufwand klären und in die Wege leiten – was nicht nur die Organisation der Pflege erleichtert, sondern auch die anderen pflegenden Familienmitglieder entlastet.	*<Ich bin schon bewusster damit um einmal achte ich auf mehr auf meine Mutter wie geht es ihr […] ich achte auch mehr ja auf meine Familie […] aber auch dass ich jetzt nicht jedem erzähle dass meine Mutter dement ist aber ich merke eben dass es vielen so geht […] es is=kein Einzelfall oder n=Superschicksal von mir […] so dass man sich da also keine Vorwürfe machen müsste> (Nebenpflegender, Netzwerk A)*
